# Supplementary material for: Machine Learning Accelerates Crystallization for Structure Determination
Source: Angew Chem Int Ed Engl. 2026 May 4;65(25):e1218503. doi: 10.1002/anie.1218503 (PMC13266958; doi:10.1002/anie.1218503)

checkCIF/PLATON report

Structure factors have been supplied for datablock(s) lcz053

THIS REPORT IS FOR GUIDANCE ONLY. IF USED AS PART OF A REVIEW PROCEDURE FOR PUBLICATION, IT SHOULD NOT REPLACE THE EXPERTISE OF AN EXPERIENCED CRYSTALLOGRAPHIC REFEREE.

No syntax errors found. CIF dictionary Interpreting this report

Datablock: lcz053

Bond precision:	C-C = 0.0371 A		Wavelength=1.54184
Cell:	a=12.6875(1)	b=22.7148(2)	c=19.3275(2)
	alpha=90	beta=91.829(1)	gamma=90
Temperature:	100 K		

	Calculated	Reported
Volume	5567.23(9)	5567.23(9)
Space group	P 21	P 1 21 1
Hall group	P 2yb	P 2yb
Moiety formula	C25 H11 Ag3 F18 N6 O3, C15 H3 Ag3 F18 N6	4(C15 H3 Ag3 F18 N6), 2(C10 H8 O3)
Sum formula	C40 H14 Ag6 F36 N12 O3	C80 H28 Ag12 F72 N24 O6
Mr	2041.85	4083.70
Dx,g cm-3	2.436	2.436
Z	4	2
Mu (mm-1)	18.230	18.230
F000	3872.0	3872.0
F000’	3891.71	
h,k,lmax	16,28,24	16,28,24
Nref	23925[12272]	17516
Tmin,Tmax	0.094,0.093	0.639,1.000
Tmin’	0.015	

Correction method= # Reported T Limits: Tmin=0.639 Tmax=1.000
AbsCorr = MULTI-SCAN

Data completeness= 1.43/0.73 Theta(max)= 78.501

R(reflections)= 0.0693(16843) wR2(reflections)= 0.1782(17516)

S = 1.074 Npar= 1750

The following ALERTS were generated. Each ALERT has the format
test-name_ALERT_alert-type_alert-level.
Click on the hyperlinks for more details of the test.

Alert level A

PLAT971_ALERT_2_A Check Calcd Resid. Dens. 1.17Ang From Ag01 3.67 eA-3

Author Response: This Alert is due to presence of residual density in the presence of heavy metal atom (Ag).

PLAT971_ALERT_2_A Check Calcd Resid. Dens. 1.24Ang From Ag02 3.53 eA-3

Author Response: This Alert is due to presence of residual density in the presence of heavy metal atom (Ag).

PLAT971_ALERT_2_A Check Calcd Resid. Dens. 1.26Ang From Ag03 3.52 eA-3

Author Response: This Alert is due to presence of residual density in the presence of heavy metal atom (Ag).

PLAT971_ALERT_2_A Check Calcd Resid. Dens. 1.24Ang From Ag09 3.51 eA-3

Author Response: This Alert is due to presence of residual density in the presence of heavy metal atom (Ag).

Alert level B

PLAT342_ALERT_3_B Low Bond Precision on C-C Bonds .... 0.03712 Ang.

Author Response: Disordered structure.

PLAT915_ALERT_3_B No Flack x Check Done: Low Friedel Pair Coverage 49 %

Author Response: Due to insufficient data. The compound crystallizes in the chiral space group, but no chiral ligand is used, and the complex is no longer chiral.

```
PLAT971_ALERT_2_B Check Calcd Resid. Dens.  1.16Ang From N02N           3.47 eA-3
```

Author Response: This Alert is due to presence of residual density in the presence of heavy metal atom (Ag).

```
PLAT971_ALERT_2_B Check Calcd Resid. Dens.  1.22Ang From N01J           3.25 eA-3
```

Author Response: This Alert is due to presence of residual density in the presence of heavy metal atom (Ag).

```
PLAT971_ALERT_2_B Check Calcd Resid. Dens.  1.22Ang From Ag07           3.25 eA-3
```

Author Response: This Alert is due to presence of residual density in the presence of heavy metal atom (Ag).

```
PLAT971_ALERT_2_B Check Calcd Resid. Dens.  1.23Ang From Ag05           3.20 eA-3
```

Author Response: This Alert is due to presence of residual density in the presence of heavy metal atom (Ag).

```
PLAT971_ALERT_2_B Check Calcd Resid. Dens.  1.00Ang From N00S           3.18 eA-3
```

Author Response: This Alert is due to presence of residual density in the presence of heavy metal atom (Ag).

```
PLAT971_ALERT_2_B Check Calcd Resid. Dens.  1.23Ang From N00H           3.17 eA-3
```

Author Response: This Alert is due to presence of residual density in the presence of heavy metal atom (Ag).

```
PLAT971_ALERT_2_B Check Calcd Resid. Dens.  1.17Ang From Ag09           3.12 eA-3
```

Author Response: This Alert is due to presence of residual density in the presence of heavy metal atom (Ag).

```
PLAT971_ALERT_2_B Check Calcd Resid. Dens.  1.25Ang From N03B           3.11 eA-3
```

Author Response: This Alert is due to presence of residual density in the presence of heavy metal atom (Ag).

```
PLAT971_ALERT_2_B Check Calcd Resid. Dens.  1.26Ang From Ag05           3.01 eA-3
```

Author Response: This Alert is due to presence of residual density in the presence of heavy metal atom (Ag).

PLAT971_ALERT_2_B Check Calcd Resid. Dens. 1.07Ang From Ag0A 2.91 eA-3

Author Response: This Alert is due to presence of residual density in the presence of heavy metal atom (Ag).

PLAT971_ALERT_2_B Check Calcd Resid. Dens. 0.97Ang From N02D 2.90 eA-3

Author Response: This Alert is due to presence of residual density in the presence of heavy metal atom (Ag).

PLAT971_ALERT_2_B Check Calcd Resid. Dens. 1.26Ang From Ag08 2.87 eA-3

Author Response: This Alert is due to presence of residual density in the presence of heavy metal atom (Ag).

PLAT971_ALERT_2_B Check Calcd Resid. Dens. 1.11Ang From N00L 2.86 eA-3

Author Response: This Alert is due to presence of residual density in the presence of heavy metal atom (Ag).

PLAT971_ALERT_2_B Check Calcd Resid. Dens. 1.21Ang From Ag0C 2.78 eA-3

Author Response: This Alert is due to presence of residual density in the presence of heavy metal atom (Ag).

PLAT971_ALERT_2_B Check Calcd Resid. Dens. 1.16Ang From Ag08 2.73 eA-3

Author Response: This Alert is due to presence of residual density in the presence of heavy metal atom (Ag).

PLAT971_ALERT_2_B Check Calcd Resid. Dens. 1.18Ang From Ag04 2.71 eA-3

Author Response: This Alert is due to presence of residual density in the presence of heavy metal atom (Ag).

PLAT971_ALERT_2_B Check Calcd Resid. Dens. 1.14Ang From Ag02 2.66 eA-3

Author Response: This Alert is due to presence of residual density in the presence of heavy metal atom (Ag).

```
PLAT971_ALERT_2_B Check Calcd Resid. Dens.  1.17Ang From Ag0C           2.63 eA-3
```

Author Response: This Alert is due to presence of residual density in the presence of heavy metal atom (Ag).

```
PLAT971_ALERT_2_B Check Calcd Resid. Dens.  1.18Ang From Ag03           2.59 eA-3
```

Author Response: This Alert is due to presence of residual density in the presence of heavy metal atom (Ag).

Alert level C

```
DIFMX02_ALERT_1_C  The maximum difference density is > 0.1*ZMAX*0.75
           The relevant atom site should be identified.
PLAT041_ALERT_1_C Calc. and Reported SumFormula    Strings  Differ     Please Check
              Calc: C40 H14 Ag6 F36 N12 O3
              Rep.: C80 H28 Ag12 F72 N24 O6
PLAT042_ALERT_1_C Calc. and Reported MoietyFormula Strings  Differ     Please Check
              Calc: C25 H11 Ag3 F18 N6 O3, C15 H3 Ag3 F18 N6
              Rep.: 4(C15 H3 Ag3 F18 N6), 2(C10 H8 O3)
PLAT090_ALERT_3_C Poor Data / Parameter Ratio (Zmax > 18) ........       6.73 Note
PLAT094_ALERT_2_C Ratio of Maximum / Minimum Residual Density ....       3.21 Report
PLAT097_ALERT_2_C Large Reported Max.  (Positive) Residual Density       3.72 eA-3
PLAT213_ALERT_2_C Atom F045            has ADP max/min Ratio .....        3.3 prolat
PLAT213_ALERT_2_C Atom C03W            has ADP max/min Ratio .....        3.1 oblate
PLAT213_ALERT_2_C Atom F00Q            has ADP max/min Ratio .....        3.5 oblate
PLAT213_ALERT_2_C Atom F01Z            has ADP max/min Ratio .....        3.3 prolat
PLAT213_ALERT_2_C Atom F021            has ADP max/min Ratio .....        3.4 prolat
PLAT213_ALERT_2_C Atom F02V            has ADP max/min Ratio .....        3.4 prolat
PLAT213_ALERT_2_C Atom F02X            has ADP max/min Ratio .....        3.6 oblate
PLAT213_ALERT_2_C Atom O02P            has ADP max/min Ratio .....        3.1 prolat
PLAT213_ALERT_2_C Atom C039            has ADP max/min Ratio .....        3.6 oblate
PLAT213_ALERT_2_C Atom C04X            has ADP max/min Ratio .....        3.6 prolat
PLAT213_ALERT_2_C Atom C04Y            has ADP max/min Ratio .....        3.5 prolat
PLAT220_ALERT_2_C NonSolvent   Resd 1  C   Ueq(max)/Ueq(min) Range        4.5 Ratio
PLAT220_ALERT_2_C NonSolvent   Resd 1  F   Ueq(max)/Ueq(min) Range        4.6 Ratio
PLAT220_ALERT_2_C NonSolvent   Resd 2  C   Ueq(max)/Ueq(min) Range        3.8 Ratio
PLAT222_ALERT_3_C NonSolvent Resd 1  H   Uiso(max)/Uiso(min) Range        5.0 Ratio
PLAT234_ALERT_4_C Large Hirshfeld Difference F00D     --C02S     .       0.16 Ang.
PLAT234_ALERT_4_C Large Hirshfeld Difference F056     --C051     .       0.20 Ang.
PLAT234_ALERT_4_C Large Hirshfeld Difference O00T     --C04S     .       0.18 Ang.
PLAT234_ALERT_4_C Large Hirshfeld Difference F057     --C05A     .       0.22 Ang.
PLAT234_ALERT_4_C Large Hirshfeld Difference O02P     --C05C     .       0.22 Ang.
PLAT234_ALERT_4_C Large Hirshfeld Difference C01Y     --C050     .       0.20 Ang.
PLAT234_ALERT_4_C Large Hirshfeld Difference Ag0B     --N02D     .       0.18 Ang.
PLAT234_ALERT_4_C Large Hirshfeld Difference N01W     --N02D     .       0.20 Ang.
PLAT241_ALERT_2_C High   'MainMol' Ueq as Compared to Neighbors of       C04M Check
PLAT241_ALERT_2_C High   'MainMol' Ueq as Compared to Neighbors of       N02D Check
PLAT241_ALERT_2_C High   'MainMol' Ueq as Compared to Neighbors of       C030 Check
PLAT362_ALERT_2_C Short  C(sp3)-C(sp2) Bond  C02W     - C03K     .       1.39 Ang.
PLAT369_ALERT_2_C Long   C(sp2)-C(sp2) Bond  C035     - C04E     .       1.53 Ang.
PLAT911_ALERT_3_C Missing FCF Refl Between Thmin & STh/L=    0.600         16 Report
```

```
                 0   2   0,   11  18   3,    0  11   4,   -6   0   5,   -7   2   5,    4  22  12,
               -10  13  14,    9   9  16,    9  10  16,    6   0  21,    2   9  21,    2  10  21,
                 4   0  22,   -1   0  23,    0   0  23,   -1   1  23,
PLAT971_ALERT_2_C Check Calcd Resid. Dens.  1.06Ang From Ag01           2.27 eA-3
```

Author Response: This Alert is due to presence of residual density in the presence of heavy metal atom (Ag).

```
PLAT971_ALERT_2_C Check Calcd Resid. Dens.  1.04Ang From Ag05           2.19 eA-3
```

Author Response: This Alert is due to presence of residual density in the presence of heavy metal atom (Ag).

```
PLAT977_ALERT_2_C Check Negative Difference Density on H03H      .      -0.44 eA-3
PLAT977_ALERT_2_C Check Negative Difference Density on H03I      .      -0.58 eA-3
PLAT977_ALERT_2_C Check Negative Difference Density on H04U      .      -0.63 eA-3
```

Alert level G

```
PLAT003_ALERT_2_G Number of Uiso or U(i,j)  Restrained non-H-Atoms         47 Report
PLAT045_ALERT_1_G Calculated and Reported Z Differ by a Factor ...          2 Check
PLAT083_ALERT_2_G SHELXL Second Parameter in WGHT  Unusually Large     204.54 Why ?
PLAT142_ALERT_4_G s.u. on b - Axis Small or Missing .............    0.00020 Ang.
PLAT177_ALERT_4_G The CIF-Embedded .res File Contains DELU Records          5 Report
PLAT186_ALERT_4_G The CIF-Embedded .res File Contains ISOR Records          6 Report
PLAT192_ALERT_3_G A Non-default DELU Restraint Value for SecondPar     0.0200 Report
PLAT192_ALERT_3_G A Non-default DELU Restraint Value for SecondPar     0.0200 Report
PLAT192_ALERT_3_G A Non-default DELU Restraint Value for SecondPar     0.0200 Report
PLAT192_ALERT_3_G A Non-default DELU Restraint Value for SecondPar     0.0200 Report
PLAT192_ALERT_3_G A Non-default DELU Restraint Value for SecondPar     0.0200 Report
PLAT242_ALERT_2_G Low    'MainMol' Ueq as Compared to Neighbors of       C02S Check
PLAT242_ALERT_2_G Low    'MainMol' Ueq as Compared to Neighbors of       C04C Check
PLAT242_ALERT_2_G Low    'MainMol' Ueq as Compared to Neighbors of       C04T Check
PLAT242_ALERT_2_G Low    'MainMol' Ueq as Compared to Neighbors of       C051 Check
PLAT242_ALERT_2_G Low    'MainMol' Ueq as Compared to Neighbors of       C058 Check
PLAT242_ALERT_2_G Low    'MainMol' Ueq as Compared to Neighbors of       C059 Check
PLAT242_ALERT_2_G Low    'MainMol' Ueq as Compared to Neighbors of       C02R Check
PLAT242_ALERT_2_G Low    'MainMol' Ueq as Compared to Neighbors of       C03V Check
PLAT242_ALERT_2_G Low    'MainMol' Ueq as Compared to Neighbors of       C04Q Check
PLAT242_ALERT_2_G Low    'MainMol' Ueq as Compared to Neighbors of       C050 Check
PLAT242_ALERT_2_G Low    'MainMol' Ueq as Compared to Neighbors of       C052 Check
PLAT242_ALERT_2_G Low    'MainMol' Ueq as Compared to Neighbors of       C05A Check
PLAT242_ALERT_2_G Low    'MainMol' Ueq as Compared to Neighbors of       C02W Check
PLAT242_ALERT_2_G Low    'MainMol' Ueq as Compared to Neighbors of       C04A Check
PLAT242_ALERT_2_G Low    'MainMol' Ueq as Compared to Neighbors of       C04D Check
PLAT242_ALERT_2_G Low    'MainMol' Ueq as Compared to Neighbors of       C04R Check
PLAT242_ALERT_2_G Low    'MainMol' Ueq as Compared to Neighbors of       C053 Check
PLAT242_ALERT_2_G Low    'MainMol' Ueq as Compared to Neighbors of       C055 Check
PLAT242_ALERT_2_G Low    'MainMol' Ueq as Compared to Neighbors of       C03P Check
PLAT242_ALERT_2_G Low    'MainMol' Ueq as Compared to Neighbors of       C03X Check
PLAT242_ALERT_2_G Low    'MainMol' Ueq as Compared to Neighbors of       C047 Check
PLAT242_ALERT_2_G Low    'MainMol' Ueq as Compared to Neighbors of       C04F Check
PLAT242_ALERT_2_G Low    'MainMol' Ueq as Compared to Neighbors of       C04P Check
PLAT434_ALERT_2_G Short Inter HL..HL Contact F010     ..F04V     .       2.79 Ang.
```

2-x,1/2+y,2-z = 2_757 Check
PLAT434_ALERT_2_G Short Inter HL..HL Contact F01E ..F034 . 2.80 Ang.
x,y,z = 1_555 Check
PLAT434_ALERT_2_G Short Inter HL..HL Contact F02A ..F057 . 2.84 Ang.
x,y,z = 1_555 Check
PLAT720_ALERT_4_G Number of Unusual/Non-Standard Labels .... 222 Note

Ag01	Ag02	Ag03	Ag04	Ag05	Ag06	Ag07	Ag08
Ag09	Ag0A	Ag0B	Ag0C	F00D	F00E	F00F	F00G
N00H	F00I	N00J	N00K	N00L	F00M	F00N	F00O
N00P	F00Q	N00R	N00S	O00T	F00U	F00V	N00W
N00X	F00Y	N00Z	F010	F011	F012	F013	F014
F015	F016	N017	N018	F019	F01A	N01B	F01C
N01D	F01E	F01F	F01G	F01H	F01I	N01J	F01K
F01L	F01M	O01N	O01O	O01P	F01Q	N01R	F01S
F01T	C01U	N01V	N01W	F01X	C01Y	F01Z	F020
F021	N022	F023	C024	C025	H025	F026	N027
F028	F029	F02A	C02B	H02B	F02C	N02D	N02E
C02F	C02G	H02G	F02H	F02I	C02J	C02K	H02K
F02L	C02M	N02N	F02O	O02P	C02Q	C02R	C02S
F02T	F02U	F02V	C02W	F02X	F02Y	C02Z	C030
H030	C031	C032	H032	F033	F034	C035	C036
H036	F037	C038	C039	H039	C03A	H03A	N03B
F03C	C03D	C03E	C03F	C03G	C03H	H03H	C03I
H03I	F03J	C03K	F03L	C03M	H03M	C03N	H03N
C03O	C03P	C03Q	C03R	C03S	H03S	F03T	F03U
C03V	C03W	H03W	C03X	F03Y	C03Z	C040	C041
H041	O042	C043	C044	F045	C046	C047	F048
C049	C04A	C04B	H04B	C04C	C04D	C04E	H04E
C04F	F04G	C04H	F04I	C04J	H04J	C04K	F04L
C04M	H04M	C04N	C04O	C04P	C04Q	C04R	C04S
C04T	C04U	H04U	F04V	F04W	C04X	C04Y	H04Y
C04Z	C050	C051	C052	C053	C054	C055	F056
F057	C058	C059	C05A	C05B	H05A	H05B	H05C
C05C	H05D	H05E	H05F	F05F	F05G		

PLAT722_ALERT_1_G Angle Calc 122.00, Rep 123.30 Dev... 1.30 Degree
C04Z -C04M -H04M 1_555 1_555 1_555 # 298 Check
PLAT790_ALERT_4_G Centre of Gravity not Within Unit-Cell: Resd. # 3 Note
C15 H3 Ag3 F18 N6
PLAT860_ALERT_3_G Number of Least-Squares Restraints .... 258 Note
PLAT883_ALERT_1_G Absent Datum for _atom_sites_solution_primary .. Please Do !
PLAT910_ALERT_3_G Missing FCF Reflection(s) Below Theta(Min)[Deg]= 3.00 Note
0 0 1,
PLAT912_ALERT_4_G Missing # of FCF Reflections Above STh/L= 0.600 481 Note
PLAT930_ALERT_2_G FCF-based Twin Law (0 0 1) Est.d BASF 0.17 Check
PLAT931_ALERT_5_G CIFcalcFCF Twin Law (0 0 1) Est.d BASF 0.17 Check
PLAT941_ALERT_3_G Average HKL Measurement Multiplicity .... 3.0 Low
PLAT969_ALERT_5_G The 'Henn et al.' R-Factor-gap value .... 3.396 Note
Predicted wR2: Based on SigI**2 5.25 or SHELX Weight 16.60
PLAT978_ALERT_2_G Number C-C Bonds with Positive Residual Density. 0 Info

---

4 **ALERT level A** = Most likely a serious problem - resolve or explain
21 **ALERT level B** = A potentially serious problem, consider carefully
40 **ALERT level C** = Check. Ensure it is not caused by an omission or oversight
49 **ALERT level G** = General information/check it is not something unexpected

6 ALERT type 1 CIF construction/syntax error, inconsistent or missing data

```
 79 ALERT type 2 Indicator that the structure model may be wrong or deficient
 13 ALERT type 3 Indicator that the structure quality may be low
 14 ALERT type 4 Improvement, methodology, query or suggestion
  2 ALERT type 5 Informative message, check
```

It is advisable to attempt to resolve as many as possible of the alerts in all categories. Often the minor alerts point to easily fixed oversights, errors and omissions in your CIF or refinement strategy, so attention to these fine details can be worthwhile. It is up to the individual to critically assess their own results and, if necessary, seek expert advice.

Validation response form

Please find below a validation response form (VRF) that can be filled in and pasted into your CIF.

```
# start Validation Reply Form
_vrf_DIFMX02_lcz053
;
PROBLEM: The maximum difference density is > 0.1*ZMAX*0.75
RESPONSE: ...
;
_vrf_PLAT041_lcz053
;
PROBLEM: Calc. and Reported SumFormula    Strings  Differ     Please Check
RESPONSE: ...
;
_vrf_PLAT042_lcz053
;
PROBLEM: Calc. and Reported MoietyFormula Strings  Differ     Please Check
RESPONSE: ...
;
_vrf_PLAT090_lcz053
;
PROBLEM: Poor Data / Parameter Ratio (Zmax > 18) ........       6.73 Note
RESPONSE: ...
;
_vrf_PLAT094_lcz053
;
PROBLEM: Ratio of Maximum / Minimum Residual Density ....       3.21 Report
RESPONSE: ...
;
_vrf_PLAT097_lcz053
;
PROBLEM: Large Reported Max.  (Positive) Residual Density       3.72 eA-3
RESPONSE: ...
;
_vrf_PLAT213_lcz053
;
PROBLEM: Atom F045            has ADP max/min Ratio .....        3.3 prolat
RESPONSE: ...
;
_vrf_PLAT220_lcz053
;
PROBLEM: NonSolvent   Resd 1  C   Ueq(max)/Ueq(min) Range        4.5 Ratio
```

```
RESPONSE: ...
;
_vrf_PLAT222_lcz053
;
PROBLEM: NonSolvent Resd 1  H   Uiso(max)/Uiso(min) Range        5.0 Ratio
RESPONSE: ...
;
_vrf_PLAT234_lcz053
;
PROBLEM: Large Hirshfeld Difference F00D     --C02S     .       0.16 Ang.
RESPONSE: ...
;
_vrf_PLAT241_lcz053
;
PROBLEM: High   'MainMol' Ueq as Compared to Neighbors of       C04M Check
RESPONSE: ...
;
_vrf_PLAT362_lcz053
;
PROBLEM: Short  C(sp3)-C(sp2) Bond  C02W     - C03K     .       1.39 Ang.
RESPONSE: ...
;
_vrf_PLAT369_lcz053
;
PROBLEM: Long   C(sp2)-C(sp2) Bond  C035     - C04E     .       1.53 Ang.
RESPONSE: ...
;
_vrf_PLAT911_lcz053
;
PROBLEM: Missing FCF Refl Between Thmin & STh/L=    0.600         16 Report
RESPONSE: ...
;
_vrf_PLAT977_lcz053
;
PROBLEM: Check Negative Difference Density on H03H      .      -0.44 eA-3
RESPONSE: ...
;
# end Validation Reply Form
```

PLATON version of 17/09/2025; check.def file version of 17/09/2025

---

duplicate check

No duplication found

Datablock lcz053 - ellipsoid plot

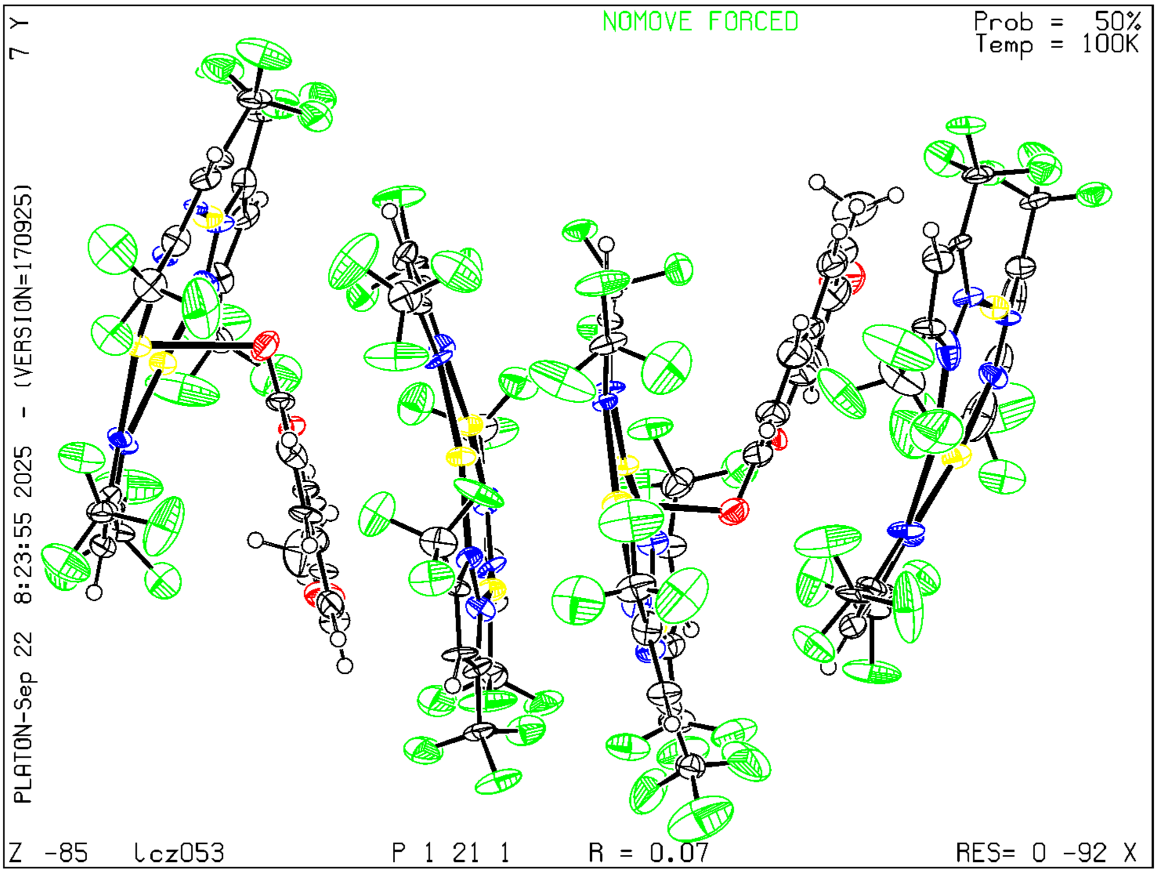

Supplement: Supplementary file 2 — Supporting File 2: anie72462‐sup‐0002‐DataFile.zip. [file ANIE-65-e1218503-s002.zip › supplementary_materials_crystallographic_data submit.zip!/cif_and_checkcif(1-114)/Ag3Pz3_53/Ag3Pz3_53_checkcif.pdf]
